# Factors Affecting 25-Hydroxyvitamin D Concentration in Response to Vitamin D Supplementation

**DOI:** 10.3390/nu7075111

**Published:** 2015-06-25

**Authors:** Hajar Mazahery, Pamela R. von Hurst

**Affiliations:** Institute of Food Science and Technology–School of Food and Nutrition, Massey University, Auckland 0632, New Zealand; E-Mail: h.mazahery@hotmail.com

**Keywords:** vitamin D, 25(OH)D, supplementation, response, review

## Abstract

Sun exposure is the main source of vitamin D. Due to many lifestyle risk factors vitamin D deficiency/insufficiency is becoming a worldwide health problem. Low 25(OH)D concentration is associated with adverse musculoskeletal and non-musculoskeletal health outcomes. Vitamin D supplementation is currently the best approach to treat deficiency and to maintain adequacy. In response to a given dose of vitamin D, the effect on 25(OH)D concentration differs between individuals, and it is imperative that factors affecting this response be identified. For this review, a comprehensive literature search was conducted to identify those factors and to explore their significance in relation to circulating 25(OH)D response to vitamin D supplementation. The effect of several demographic/biological factors such as baseline 25(OH)D, aging, body mass index(BMI)/body fat percentage, ethnicity, calcium intake, genetics, oestrogen use, dietary fat content and composition, and some diseases and medications has been addressed. Furthermore, strategies employed by researchers or health care providers (type, dose and duration of vitamin D supplementation) and environment (season) are other contributing factors. With the exception of baseline 25(OH)D, BMI/body fat percentage, dose and type of vitamin D, the relative importance of other factors and the mechanisms by which these factors may affect the response remains to be determined.

## 1. Introduction

According to the Endocrine Society’s definition of vitamin D insufficiency and deficiency, 25(OH)D levels of 50–75 and below 50 nmol/L, respectively [1], it has been estimated that vitamin D insufficiency affects one billion people around the world [2]. The prevalence of vitamin D deficiency in the US, Australian and New Zealand populations has been reported to be 27%–32% [3,4,5]. While vitamin D insufficiency has been reported to be common throughout the world [5], vitamin D deficiency is more prevalent in certain regions and ethnic groups [3]. It should be noted that depending on the definitions used by different scientific societies, the prevalence of vitamin D deficiency and insufficiency varies drastically.

Traditionally, severe vitamin D deficiency, deficiency and insufficiency were defined as 25(OH)D concentrations <12, <25 and 25–50 nmol/L, accordingly [6]. There is a consensus between Ministry of Health and Cancer Society of New Zealand [7], Institute of Medicine [8] and American Academy of Dermatology (AAD) and AAD association [9] that the minimum 25(OH)D concentrations for a better health outcome are at least 50 nmol/L. However, the Endocrine Society in the US proposed concentrations of 75 nmol/L or more for multiple clinical outcomes [1]. For the purpose of this review and to avoid confusion, the concentrations of 25(OH)D are reported instead of vitamin D “deficiency or insufficiency” unless the cutoffs are otherwise stated.

To prevent or combat vitamin D deficiency/insufficiency, vitamin D supplementation is an easy, effective and cost-effective strategy. However, in response to a given dose of vitamin D supplement, the increase in 25(OH)D concentration has been reported to differ between individuals [10,11,12,13,14]. Because of the wide inter-individual variation [15], the one-size-fits-all approach does not work with vitamin D supplementation, and it is imperative that clinicians take those factors affecting the response to vitamin D supplements into account and individualize their strategy.

Response to vitamin D supplementation can be explained by several environmental and demographic factors. Recently, Zittermann *et al*. (2014) [16] published a systematic review concerning the importance of body weight for the dose-response relationship with circulating 25(OH)D. The authors demonstrated that 34.5% of variation in circulating 25(OH)D was explained by body weight, followed by type of supplement (D_2_ or D_3_) (9.8%), age (3.7%), calcium intake (2.4%) and basal 25(OH)D concentrations (1.9%), leaving approximately 50% of the variations to unknown factors.

For these reasons, we aimed to investigate possible factors and to examine their significance in relation to circulating 25(OH)D response to vitamin D supplementation. A comprehensive literature search in several databases (PubMed, PMC and Embase) using the following search terms: vitamin D or cholecalciferol or ergocalciferol and supplementation was performed. Intervention trials that reported information on circulating 25(OH)D concentration at baseline and follow-up and reported data on factors predicting/affecting 25(OH)D response to vitamin D supplementation were considered. Studies in children and in patients with conditions that affect vitamin D metabolism, such as chronic kidney diseases were excluded. Herein, we first present an overview of vitamin D metabolism, biomarkers and roles in the body and then present the results of the review.

## 2. Vitamin D: Metabolism, Biomarkers and Roles in the Body

Sun exposure/UVB radiation on skin is the most common and efficient source of vitamin D [17]. Vitamin D is also naturally present in very few foods, added to others and available as a dietary supplement [17]. Vitamin D either ingested from diet or from UVB-induced conversion of 7-dehydrocholesterol in the skin undergoes enzymatic hydroxylation (25-hydroxylase) in the liver and forms 25(OH)D [18]. This metabolite, 25(OH)D, is the major circulating form of vitamin D, and is metabolically inactive until it is converted to 1,25-dihydroxyvitamin D [1,25(OH)2D] by an enzymatic hydroxylation process (25-hydroxyvitamin D-1α-hydroxylase) [2]. The active metabolite, 1,25(OH)_2_D, acting through vitamin D receptors (VDR) can produce a wide range of skeletal and non-skeletal effects [6,19]. 1,25(OH)_2_D acts either synergistically with parathyroid hormone (PTH) or alone and modulates calcium homeostasis and bone metabolism. 1,25(OH)_2_D increases serum calcium concentrations by increasing skeletal mobilisation of calcium, renal calcium re-absorption [18] and intestinal calcium absorption [20]. Evidence in the laboratory also indicates that 1,25(OH)_2_D_3_ has a number of non-skeletal effects including, inhibition of autoimmune diseases [21] and cancer progression [22,23], modulation of immune system [24], and regulation of cardiovascular system [25] and adipocyte apoptosis [23,26].

The system of vitamin D metabolism acts according to the first-order reaction enzyme kinetics [27]. When vitamin D supplies are low, the enzymatic capacity of non-renal tissues to produce 1,25(OH)_2_D is diminished. Accordingly, flow of 25(OH)D through other potential pathways is compromised to maintain the circulating concentration of 1,25(OH)_2_D at the level determined by the priority requirements of calcium homeostasis and bone metabolism. In contrast, under conditions of adequate supply of vitamin D, higher 25(OH)D concentrations meet all physiological requirements for both skeletal and non-skeletal pathways on one hand, and up-regulate 24-hydroxylase and the catabolic pathways associated with it on the other hand.

More than 50 different vitamin D metabolites have been identified, of which vitamin D, 1,25(OH)_2_D and 25(OH)D have been the focus of vitamin D assay methods [28]. Circulating 25(OH)D is currently considered the best determinant of vitamin D status compared to other vitamin D metabolites namely vitamin D and 1,25(OH)_2_D because: (1) its half-life is much longer, though the definite time is controversial (ranging from three weeks to three months) [29,30]; (2) its production in the liver is not significantly regulated and depends on the substrate availability [28] and (3) its concentration reflects body stores of both vitamin D synthesized in the skin and the vitamin D ingested from a diet or supplement. Vitamin D [28] and 1,25(OH)_2_D [29], in contrast, have a short half-life of 24 h and 4 to 6 h, respectively. Compared to 25(OH)D, serum concentration of 1,25(OH)_2_D is very low (about a thousand-fold less) [29] and its production is tightly regulated by a person’s calcium requirements [28].

Not only is circulating 25(OH)D an indicator of vitamin D status, but it is also a marker of good health. Serum 25(OH)D concentrations <25 nmol/L are associated with decreased intestinal calcium absorption [31], though a minimum level of 75 nmol/L has been proposed for optimal calcium absorption by Barger-Lux and Heaney (2002) [32]. It is generally recognised that prolonged and severe vitamin D deficiency (25(OH)D < 20 nmol/L) is associated with the symptoms and signs of rickets in children and osteomalacia in adults [33], albeit higher 25(OH)D levels are required to ensure multiple health outcomes. Evidence shows that circulating 25(OH)D levels >75 nmol/L are associated with decreased risk of cardiovascular diseases [34], decreased activity and progression of multiple sclerosis [35] and increased survival in patients with colorectal and breast cancer [36]. The high cost of treating patients with these diseases is an escalating public health problem, possibly exacerbated as the prevalence of the circulating levels of 25(OH)D below 75 nmol/L (as a risk factor) continues to increase.

## 3. Factors Affecting Circulating 25(OH)D Concentration in Response to Vitamin D Supplementation

There are many factors which can influence the way individuals respond to, and metabolize supplemental vitamin D. From the available evidence, we categorized factors into two groups; (1) factors associated with the individual characteristics and biological parameters; and (2) factors associated with environment and treatment strategy. All factors within each category will be discussed in more detail in the following sections.

### 3.1. Biological and Demographic Characteristics Determinants

Several biological and demographic factors, including baseline 25(OH)D, age, BMI or body fat percentage, ethnicity and calcium intake, have been well examined in relation to the response to vitamin D supplementation (Table 1). However, other variables, such as genetics, oestrogen use, dietary fat content and composition and some diseases and medications have been investigated to a lesser extent. In this section the evidence for these potential determinants will be examined.

#### 3.1.1. Basal 25(OH)D Concentration

Baseline 25(OH)D concentration has been consistently shown to make a significant contribution to variance in 25(OH)D response to vitamin D supplementation (Table 1) [10,14,15,37,38,39,40,41,42,43,44,45,46,47,48,49,50]. Because hepatic hydroxylation of vitamin D may be a saturable process [40], response to vitamin D supplementation could well be affected by baseline 25(OH)D concentrations. Baseline 25(OH)D concentration explained 20.2% of the variation in 25(OH)D response to vitamin D supplementation in a cohort of Middle Eastern women (*n* = 62) [15]. In response to supplementation with daily 4000 IU vitamin D for 14 days, Trang *et al*. (1998) showed that change in 25(OH)D concentration had a significant inverse correlation with baseline 25(OH)D concentrations [44]. The largest increase was seen in subjects in the first tertile (10–34 nmol/L), followed by those in the second tertile (35–49 nmol/L) and then those in the third tertile (50–86 nmol/L); +30.6 ± 16.2, +25.5 ± 11.7 and +13.3 ± 13.9 nmol/L, respectively (*p* = 0.02).

Bacon *et al*. (2009) demonstrated that deficient subjects (<50 nmol/L) receiving a loading dose of 500,000 IU had larger incremental change in their 25(OH)D concentrations at one month than non-deficient subjects (≥50 nmol/L), 71.0 [95% CI, 58.0–84.0] *vs.* 50.0 [95% CI, 38.0–63.0] nmol/L (*p* = 0.03), respectively [43]. Similarly, Canto-Costa *et al*. (2006) found that while the mean increase was 25.4 nmol/L in subjects with 25(OH)D concentrations <50 nmol/L, it was 13.0 nmol/L in those with 25(OH)D concentrations >50 nmol/L (*p* < 0.05). The participants were housebound elderly men and women (*n* = 42) and received weekly 7000 IU vitamin D_3_ supplements for 12 weeks [37].

**Table 1:** Demographic and biological factors predicting circulating 25(OH)D response to vitamin D supplementation.

Study	Population Characteristics	Study Design/Duration/Groups	Relationship with						Description
			Basal 25(OH)D	Age	BMI/weight	Body Fat %	Ethnicity	Calcium Intake	
Aloia *et al*. (2008) [10]	Healthy men and women (*n* = 138)	Randomised double blind placebo control trial/6 months//Dosing at baseline started with daily 2000 IU D_3_ and daily 4000 IU D_3_ for those with >50 and ≤50 nmol/L, respectively. Then, the intake was modified.	**Y**	**N**	**N**	**N**	N*		Inverse relationship with basal 25(OH)D. * No significant racial differences in response to supplementation. But, African Americans needed higher doses than white Americans to achieve 25(OH)D concentrations of 75 nmol/L or more by 18 weeks (+50%).
Bacon *et al.* (2009) [43]	Elderly men and women (*n* = 63)	Randomised double blind trial/8 months/Single dose of 500,000 IU (loading dose), loading dose + monthly 50,000 IU or monthly 50,000 IU	**Y**						At one month, larger increase in 25(OH)D concentrations was seen in deficient subjects compared to non-deficient subject
Barger-Lux *et al.* (1998) [40]	Healthy men (*n* = 116)	Open labelled trial/8 weeks/daily 1000, 10,000, or 50,000 IU D_3_ or other vitamin D metabolites	**Y**		**Y**				Baseline and BMI were significant predictors of 25(OH)D concentrations and were inversely associated with response.
Bell, Shaw and Turner (1987) [51]	Healthy adults (*n* = 8)	Intervention trial/daily 100,000 IU D_3_ for 4 days and then daily 100,000 IU D_3_ + daily 2000 mg calcium for 4 days.						**Y**	Vitamin D alone increased 25(OH)D concentrations by 133% but vitamin D + calcium resulted in an increment of 63% (*p* < 0.02).
Blum *et al.* (2008) [49]	Healthy ambulatory men and women (*n* = 257)	Randomised placebo control trial/12 months/daily placebo or daily 700 IU D_3_+ daily 500 mg calcium	**Y**		**Y**				Inverse relationship with basal 25(OH)D. Mean adjusted 25(OH)D were 57.0 ± 14.0 and 40.8 ± 5.3 nmol/L in those with BMI < 25 and ≥ 30 kg/m², respectively. The adjusted change was 20% less in ≥30 compared to 25 kg/m² group.
Canto-Costa *et al.* (2006) [37]	Homebound elderly men and women (*n* = 42)	Prospective control intervention trial/12 weeks/weekly 7000 IU D_3_	**Y**			**N**			Those with serum levels <50 nmol/L had a mean increase of 25.4 nmol/L *vs.* 13.0 nmol/L in those with serum levels >50 nmol/L
DeLappe *et al.* (2006) [48]	Women (*n* = 114)	Prospective cohort intervention trail/3 months/daily 800 IU D_3_ + daily 1000 mg calcium	**Y**						The mean 25(OH)D concentration increased from 28.9 ± 11.9 and 73.9 ± 25.2 nmol/L to 52.5 ± 26.4 and 76.1 ± 22.5 nmol/L at the follow up in insufficient and sufficient subjects, respectively.
Fu *et al.* (2009) [52]	Healthy adults (*n* = 98)	Open label un-blinded intervention trial/12 months/daily 600 or 4000 IU D_3_		**N**	**N**				
Gallagher *et al.* (2012) [11]	Healthy postmenopausal women with vitamin D insufficiency (*n* = 163)	Randomised placebo control trial/12 months/daily placebo or daily 400, 800, 1600, 2400, 3200, 4000 or 4800 IU D_3_ + daily 1200–1400 mg calcium			**Y**				At 12 months, 25(OH)D concentration was higher in normal weight than overweight (a difference of 12.2 nmol/L) and obese subjects (a difference of 17.7 nmol/L).
Gallagher *et al.* (2013) [53]	Healthy postmenopausal women with vitamin D insufficiency (*n* = 110)	Randomised double blind placebo control trial/12 months/daily placebo or daily 800, 1600, 2400 and 4800 IU D_3_ + daily 1200–1400 mg calcium			**Y**		**N**	**Y**	1000 IU increase in the dose resulted in 13.0 and 10.3 nmol/L increase in 25(OH)D concentration in those with BMI < 30 and BMI ≥ 30 kg/m², respectively. The slope of dose-response was 2.9 nmol/L higher in BMI < 30 than BMI ≥ 30 kg/m². 1000 mg increase in calcium intake was associated with 9.5 nmol/L increase in 25(OH)D concentration.
Giusti *et al.* (2010) [54]	Community-dwelling elderly women with secondary hyperparathyroidism and vitamin D deficiency (*n* = 59)	Randomised control trial/6 months/300,000 IU D_3_ every 3 months or daily 1000 IU D_3_ + daily 1500 mg calcium in all groups	**N**		**Y**				BMI explained 10% of variation in 25(OH)D response to supplementation
Goussous *et al.* (2005) [42]	Healthy ambulatory men and postmenopausal women (*n* = 52)	Randomised placebo trail/3 month/daily 800 IU D_3_ (all subjects) + daily 2 × 500 mg calcium or placebo	**Y**					**N**	Inverse relationship with basal 25(OH)D (*p* < 0.001). No significant difference in 25(OH)D change between calcium and control group (calcium intake had no effect on response)
Harris *et al.* (2002) [39]	Healthy young and old men (*n* = 50)	Randomised control trail/8 weeks/daily 800 IU D_3_	**Y**	**N**					Inverse relationship with basal 25(OH)D.
Mazahery, Stonehouse and von Hurst (2015) [15]	Healthy premenopausal women (*n* = 61)	Randomised double blind placebo control trial/6 months/monthly placebo or monthly 50,000 or 100,000 IU	**Y**			**Y**			For each decrease of one unit in basal 25(OH)D and body fat percentage, the change in 25(OH)D is expected to increase by 0.6 and 0.7 nmol/L, respectively.
Nelson *et al.* (2009) [41]	Healthy pre-menopausal women (*n* = 112)	Randomised double blind placebo trial/21 weeks/daily placebo or daily 800 IU D_3_	**Y**			**Y**			Achieving optimal 25(OH)D concentrations in the winter was seen in those with higher baseline serum levels (67.4 ± 22.8 *vs.* 40.9 ± 16.4 nmol/L) and lower percent body fat (29.9% ± 7.1% *vs.* 35.4% ± 7.4%).
Ng *et al.* (2014) [47]	Healthy adults (*n* = 292)	Randomised double blind placebo control trial/3 months/daily placebo or daily 1000, 2000 or 4000 IU D_3_	**Y**	Y*	Y*				Many subgroups with a greater response had lower basal 25(OH)D. * Age and BMI were significant predictors of 25(OH)D at 3 months; the predictors of the change were not reported.
Nimitphong *et al.* (2013) [55]	Healthy adults (*n* = 39)	Un-blinded randomised control trial/3 months/daily 400 IU D_2_ or daily 400 IU D_3_ plus daily 675 mg calcium in both groups			**N**				
Putman *et al.* (2013) [56]	Healthy adolescents with vitamin D sufficiency (*n* = 53)	Double-blind, randomised trial/11 weeks/daily 200 or 1000 IU D_3_	**N**	**N**					Basal 25(OH)D concentrations, age, prior treatment with vitamin D and compliance were significant predictors of the change in 25(OH)D concentrations over time, but when those with prior treatment were excluded no difference was detected.
Saadi *et al.* (2007) [38]	Healthy nulliparous and lactating women (*n* = 178)	Open-labelled, randomised, parallel group trial/3 months/daily 2000 IU D_2_ or monthly 60,000 IU D_2_	**Y**		**Y**				Response to supplement was inversely associated with weight and baseline 25(OH)D levels.
Talwar *et al.* (2007) [14]	Healthy postmenopausal women (*n* = 208)	Randomised placebo control trial/36 months/daily placebo or daily 800 IU D_3_ for the first 2 years and then daily 2000 IU for the third year in the vitamin D group + daily 1200–1500 mg calcium in both groups	**Y**	**N**	**N**	**N**			Response to supplement was inversely associated with baseline 25(OH)D levels
Thomas, Need and Nordin (2010) [57]	Healthy postmenopausal women (*n* = 22)	Intra- and inter-subject comparison/8 weeks/daily 1000 mg calcium for one week followed by daily 1000 IU D_3_ + daily 1000 mg calcium for 7 weeks or daily 1000 IU D_3_ for 7 weeks followed by daily 1000 IU D_3_ + daily 1000 mg calcium for one week.						**Y**	Supplementation with 1000 mg calcium for one week with additional 1000 IU vitamin D daily for 7 weeks raised the mean 25(OH)D concentration more effectively than vitamin D or calcium (*p* < 0.001).
Trang *et al.* (1998) [44]	Healthy men and women (*n* = 72)	Randomised double blind trial/14 days/daily 4000 IU D_2_ or daily 4000 IU D_3_	**Y**						The largest increase was seen in subjects in the first tertile of 25(OH)D levels (10–34 nmol/L). Subjects in the third tertile (50–86 nmol/L) had lower increase in 25(OH)D concentrations compared to those in the first and second tertiles (35–49 nmol/L).
Veith *et al.* (2001) [58]	Healthy men and women (*n* = 61)	Randomised intervention trial/2–5 months/daily 1000 or 4000 IU D_3_	**N**		**N**				Response to supplement was inversely associated with weight.
Waterhouse *et al.* (2014) [46]	Healthy older adults (*n* = 385)	Randomised double blind placebo control trial/one year/monthly placebo or monthly 30,000 or 60,000 IU D_3_	**Y**	**N**	**Y**				Response to supplementation was inversely associated with basal 25(OH)D and BMI. Supplement dose and basal 25(OH)D explained 24% of variability in response to vitamin D supplementation.
Zhao *et al.* (2012) [50]	Postmenopausal women (*n* = 1063)	Randomised double blind placebo control trial/One year/daily placebo, daily 1100 IU D + daily 1400 mg calcium or daily 1400 mg calcium only	**Y**		N*				* Inconsistent results; significant inverse relationship was found when all participants were included. However, when only supplemented participants were included, BMI was not a significant predictor.
Zwart *et al.* (2011) [45]	Healthy men and women (*n* = 41)	Un-masked controlled intervention trial/6 months/daily 2000 IU or weekly 10,000 IU D (type of vitamin D was not specified)	**Y**		**Y**				Participants with lower basal 25(OH)D had a better response. Those with BMI >28 kg/m² responded poorly to treatment compared to those with BMI

These findings were confirmed by DeLappe *et al.* (2006) who supplemented women aged >65 years old with daily 800 IU vitamin D_3_ and 1000 mg calcium for three months. The mean 25(OH)D concentration increased from baseline to follow-up, but was higher in women (*n* = 36) with baseline 25(OH)D < 50 nmol/L (28.9 ± 11.9 nmol/L to 52.5 ± 26.4 nmol/L) than those women (*n* = 15) with baseline 25(OH)D ≥ 50 nmol/L who increased from 73.9 ± 25.2 nmol/L at baseline to 76.1 ± 22.5 nmol/L [48].

Of 20 studies examining the influence of basal 25(OH)D concentration on response to vitamin D supplementation (Table 1), 17 studies reported a significant relationship, while three failed to show any relationship. Those trials had small sample sizes (ranging from 53 to 61) and included participants who were all either vitamin D sufficient [56] or vitamin D deficient [54,58]. Due to the small sample size and a very narrow range of basal circulating 25(OH)D concentrations, the authors may have not had enough power to detect any differences across basal 25(OH)D groups. For example, Veith *et al.* (2001) [58] failed to show any relationship by assigning participants (*n* = 61; mean baseline 25(OH)D concentration 40.7 ± 15.4 nmol/L) to receive either 1000 IU or 4000 IU vitamin D_3_ daily for 2–5 months. In this study, the majority of participants (93.5%) had 25(OH)D concentrations <40 nmol/L.

#### 3.1.2. BMI or Body Fat Percentage

Higher body fat percentage or higher BMI have been associated with smaller increases in 25(OH)D concentrations in response to vitamin D supplementation (Table 1) [11,15,38,40,41,45,46,49,53,54]. Blum *et al.* (2008) assigned healthy ambulatory men and women aged ≥65 years to receive daily 700 IU vitamin D or placebo for one year. The change in 25(OH)D concentration was significantly inversely associated with BMI, central body fat, weight and waist circumference [49]. After one year, the mean adjusted 25(OH)D concentrations were higher in subjects with BMI <25 kg/m² than those with BMI ≥ 30 kg/m² (57.0 ± 14.0 *vs.* 40.8 ± 5.3 nmol/L, respectively) despite having comparable baseline levels. The adjusted change was 20% less in those with ≥30 compared to those in the <25 kg/m² category.

Recruiting the same age group, Gallagher *et al.* (2012) and Gallagher, Peacock, Yalamanchili, and Smith (2013) found that BMI was a significant predictor of 25(OH)D response to vitamin D supplementation in healthy postmenopausal white and African American women [11,53]. 25(OH)D concentrations were higher in normal weight than overweight (a difference of 12.2 nmol/L [95% CI, 4.2–20.2 nmol/L], *p* = 0.003) and obese women (a difference of 17.7 nmol/L [95% CI, 10.2–25.2 nmol/L], *p* < 0.001) [11]. At the 12-month time point, in African American women with BMI <30 kg/m², every 1000 IU increase in the dose resulted in a 13.0 nmol/L increase in 25(OH)D concentration while in women with BMI ≥ 30 kg/m², the same dose resulted in a 10.3 nmol/L increase. The slope of dose-response at the 12-month time point was 2.9 nmol/L higher in BMI category <30 kg/m² compared to BMI ≥ 30 kg/m² [53].

An effect of BMI and percentage body fat on 25(OH)D response to supplementation has also been reported in younger subjects [15,41,45]. Change in mean 25(OH)D concentration after 6 months, but not 3 months, was inversely associated with BMI among healthy Antarctic men and women workers with the mean age of 40.1 ± 10.0 years; those with BMI >28 kg/m² responded poorly to treatment compared to those with BMI <28 kg/m² (*p* < 0.03) [45]. Using body fat percentage as a better measure of body fat stores, Mazahery, Stonehouse and von Hurst (2015) [15] showed that for each decrease of one unit in body fat percentage, the change in 25(OH)D is expected to increase by 0.7 nmol/L.

From the available evidence one can suggest that 25(OH)D response to vitamin D supplementation declines when the BMI is more than or equal to 30 kg/m^2^. Therefore and because of many methodological considerations, some studies failed to show any relationship between anthropometric measures and response to treatment (Table 1) [14,37,50,52,55,58]. These studies have been limited by not having enough participants within different BMI categories (a mean BMI of 29.5 ± 4.0 kg/m² [14] and a standard deviation of 0.5 kg/m^2^ [52]), small sample size (*n* < 50) [37,55], using body weight instead of more reliable measures of body composition/body fat [58] and using small dose of vitamin D supplement (800 IU/day) [55]. The effect of adiposity may be more apparent when a larger dose of vitamin D is administered.

The mechanistic pathway by which adipose tissue affects circulating 25(OH)D response to vitamin D supplementation is that vitamin D is a fat soluble vitamin and is stored in body fat stores for later use [2]. The larger the volume of adipose tissue, the more likely vitamin D is trapped [59]. Experimental support for sequestration comes from human and animal studies [60]; Wortsman *et al.* (2000) [59] exposed both lean and obese individuals with comparable baseline 25(OH)D concentration to whole body UVB or 50,000 IU oral vitamin D_2_. After 24 h, 25(OH)D concentrations in obese subjects reached 57% of that in lean subjects exposed to UVB, and was inversely associated with BMI in those receiving oral vitamin D. In support of this study, a study in Wistar rats showed that following supplementation with high dose vitamin D, 25(OH)D concentration in plasma increased rapidly until it reached a plateau [60]. The plasma-25(OH)D and adipose tissue cholecalciferol accumulation occurred linearly and rapidly, and the accumulated cholecalciferol was released slowly into the circulation in the condition of energy balance. Recent evidence of sequestration of vitamin D in human adipocytes and acute increase in 25(OH)D concentrations during bariatric weight loss surgery lends credence to these observations [61].

#### 3.1.3. Aging

Aging has frequently been reported to be associated with lower levels of 25(OH)D in circulation [62,63]. It has been proposed that the capacity of the epidermis to synthesize vitamin D (due to a decrease in the precursor 7-dehydrocholesterol) [64] and the expression of vitamin D binding protein [65] is compromised by aging. However, it seems that aging has little or no effect on response to supplementation (Table 1). Comparing healthy men aged 18–35 years old with men aged 62–79 years old, Harris and Dawson-Hughes (2002) showed that supplementation with 800 IU vitamin D per day for eight weeks resulted in a significant and comparable increase in mean 25(OH)D concentrations in both age groups [39]. Other studies also reported no effect of aging on 25(OH)D response to vitamin D supplementation [10,14,46,52,56].

#### 3.1.4. Ethnicity

Vitamin D status has been consistently shown to be significantly different across different race/ethnic groups. However, the impact of ethnicity on response to vitamin D supplementation has been investigated to a lesser extent [10,53]. Aloia *et al.* (2008) [10] and Gallagher *et al.* (2013) [53] reported no difference in dose-response slopes between African Americans and white Americans. However, African Americans needed higher doses than white Americans to achieve 25(OH)D concentrations of 75 nmol/L or more by 18 weeks (+50%) which is mainly attributed to the lower baseline 25(OH)D concentrations in this ethnic group [10].

#### 3.1.5. Dietary Calcium Intake

There are very few trials examining the effect of dietary calcium intake on serum 25(OH)D response to vitamin D supplementation, and the results are mixed (Table 1). Most dose-response and efficacy trials administer calcium supplements alongside vitamin D supplements to ensure daily calcium intake of 1200–1500 mg and to minimize the confounding effect of dietary calcium intake on response to supplementation. Goussous *et al.* (2005) assigned elderly men and women with baseline calcium intake of ≤600 mg/d (diet plus supplements) to receive both 800 IU vitamin D_3_ and 1000 mg calcium or 800 IU vitamin D_3_ and placebo per day for three months [42]. Circulating 25(OH)D concentrations increased significantly in both groups, and the mean increase was comparable in both groups (+16.2 ± 14.8 nmol/L in the calcium group and +16.6 ± 17.4 nmol/L in control group, *p* > 0.05).

In another study, however, Bell, Shaw and Turner (1987) showed that the addition of 2000 mg calcium per day to daily 100,000 IU vitamin D for four days resulted in a significantly lower increase in mean 25(OH)D concentration [51]. The increment in calcium group was less than half of that observed in the control group (63% *vs.* 133%, respectively; *p* < 0.02). It should be noted that the dose of vitamin D was not anywhere near a physiologically normal dose.

Thomas, Need and Nordin (2010), in contrast, showed that supplementation with 1000 mg calcium for one week with additional 1000 IU vitamin D daily for 7 weeks raised the mean 25(OH)D concentration more effectively than vitamin D or calcium alone [57]. Similar results were reported in dose-response trials conducted to determine the effect of different dosages of vitamin D supplement on 25(OH)D concentrations [53]. Using a multivariate model, Gallagher *et al.* (2013) [53] showed that total calcium intake (diet plus supplement) was a significant covariate. Every 1000 mg increase in calcium intake was associated with a 9.5 nmol/L increase in 25(OH)D concentrations in vitamin D deficient postmenopausal African American women supplemented with vitamin D.

Increased intake of calcium is associated with a slight increase in serum calcium levels and with lower levels of serum PTH [57]. The decrease in PTH levels results in a decrease in production of 1,25(OH)_2_D by the kidneys, and an increase in the levels of 25(OH)D in the circulation [18].The increase in 25(OH)D levels could be explained by several mechanistic pathways: (1) inhibition of 25-hydroxylase by 1,25(OH)_2_D as a result of negative feedback loop (2) decrease in the use of 25(OH)D as a substrate; and (3) delayed metabolic clearance of 25(OH)D in the liver [57].

#### 3.1.6. Genetic Background

The relationship between vitamin D receptor (VDR) and vitamin D binding protein (VDBP) genotype and levels of 25(OH)D in circulation has been examined in several studies [52,55,66,67,68], though very few studies have examined the effect of VDBP genotype on 25(OH)D response to vitamin D supplementation [46,52,55]. For the purpose of this review, the effect of VDBP genotype on response to vitamin D supplementation will be discussed. In an open-label randomised intervention trial, Fu *et al.* (2009) examined the contribution of VDBP D432E and T436K SNPs to variation in 25(OH)D response to either 600 IU/day or 4000 IU/day vitamin D for one year [52]. The presence of 436 K allele was associated with lower 25(OH)D concentrations at baseline. However, the percentage increase in 25(OH)D concentration from baseline in both groups was in opposite directions; those with KK genotype had the largest increase followed by TK and then TT genotypes. In a multiple linear regression model, dose and 436 K, but not 432 E contributed significantly to overall variance, 22% (*p* < 0.0001) and 8.5% (*p* < 0.001), respectively. It should be noted that baseline 25(OH)D levels were not included in this model. The observed pattern could be due to the lower baseline 25(OH)D concentrations in carriers of 436 K allele.

Furthermore, the impact of VDBP genotype on response to vitamin D supplementation appears to be partly vitamin D-type specific. Serum-25(OH)D response to supplementation with vitamin D was examined in 39 healthy adults given 400 IU/day vitamin D_3_ or vitamin D_2_ [55]. The percentage increase in total 25(OH)D and 25(OH)D_3_ following supplementation with vitamin D_3_, but not with vitamin D_2_, was significantly affected by rs4588 genotype. Compared to CA and AA alleles, participants homozygous for GC2 allele (CC) had a significantly larger increase in 25(OH)D and 25(OH)D_3_ (5.84 ± 3.07 nmol/L for 25(OH)D and 6.09 ± 3.03 nmol/L for 25(OH)D_3_ *vs.* 22.58 ± 6.18 nmol/L for 25(OH)D (*p* < 0.01) and 22.98 ± 6.00 nmol/L for 25(OH)D_3_ (*p* < 0.01), respectively). Lack of control arm is a limitation to this study. There was also insufficient power to detect any small changes associated with vitamin D_2_ supplements due to small sample size.

#### 3.1.7. Oestrogen Use

Several cross-sectional studies have shown that oral contraceptive use may influence baseline levels of 25(OH)D but there is only one trial investigating the effect of oral contraceptives on 25(OH)D response to vitamin D supplementation [41]. Nelson *et al.* (2009) assigned healthy pre-menopausal women to receive 800 IU vitamin D or placebo for 21 weeks [41]. Factors influencing response to supplementation were treatment dose, baseline 25(OH)D, summer increase and oestrogen dose; the odds ratio for using higher dosages of oestrogen and having larger change in 25(OH)D concentrations was 1.08 (*p* = 0.01), though this difference is clinically insignificant. Possible explanation for an effect of oestrogen is that this hormone may enhance hepatic hydroxylation of vitamin D [69] and may also increase VDBP concentration in circulation [70].

#### 3.1.8. Dietary Fat Content and Fat Composition

Vitamin D is a fat soluble vitamin and it is plausible to suggest that a certain amount of fat in the diet improves its absorption. Mulligan and Licata (2010) recruited patients who were taking vitamin D supplement on an empty stomach or with a small meal but did not achieve an adequate rise in 25(OH)D concentrations (*n* = 17) [71]. The patients were instructed to take their supplements with the largest meal of day which may contain more fat. Mean 25(OH)D concentration increased by 56.7 ± 36.7% (from 76.25 ± 11.75 at baseline to 118.00 ± 27.25 nmol/L after diet modification). This trial had some limitations including its small sample size and the lack of a control group. In a systematic review evaluating the effect of the type of vehicle on vitamin D bioavailability, Grossmann *et al.* (2010) concluded that compared to vitamin D as powder or dissolved in ethanol, solubilised vitamin D in a small amount of fish oil produced greater change in 25(OH)D concentrations (mean change of 4.05, 2.75 and 0.5 nmol/L per 100 IU/day vitamin D in fish oil, powder and ethanol, respectively) [72]. It should be noted that most studies included in this review looked at 25(OH)D in circulation, but not at vitamin D bioavailability.

Looking directly at vitamin D absorption, Tangpricha *et al.* (2003) found no effect of fat content (high fat milk, low fat milk or corn oil) on vitamin D bioavailability [73]. In agreement, Niramitmahapanya *et al.* (2011) failed to show any relationship between dietary fat content and the response to supplementation [74]. The authors, however, found that fat composition was significantly associated with response to supplementation [74]. The increment in plasma-25(OH)D concentration was negatively associated with poly-unsaturated fatty acids (PUFA, *p* = 0.038), but positively with mono-unsaturated fatty acids (MUFA, *p* = 0.016) and with the ratio of MUFA/PUFA (*p* = 0.014). In contrast, a very recent randomised controlled trial showed that treatment with n-3 PUFA did not affect 25(OH)D concentrations [75].

The mechanisms by which type of fatty acids may influence vitamin D absorption are not known. Niramitmahapanya *et al.* (2011) suggested that fatty acids such as linoleic and linolenic acid may increase solubility of vitamin D in the micelles which in turn may increase the micelles’ size. As a consequence, vitamin D may stay longer in the micelles and may have difficulty in passing the intestinal mucosa [74].

#### 3.1.9. Diseases and Medications

Patients with diseases influencing the absorption and metabolism of vitamin D and those taking medications such as antiepileptic drugs, glucocorticoids, bile acid sequestrants and lipase inhibitors are more likely to have low vitamin D status. A significant proportion of people with cystic fibrosis (35.5 ± 10.1 nmol/L) [76], celiac disease (44.5 ± 18.0 nmol/L) [77], and Crohn’s disease (56.9 ± 23.7 nmol/L) [78,79] have 25(OH)D concentrations ≤75 nmol/L, suggesting that these conditions inhibit absorption of nutrients, so greater doses of vitamin D may be required. Yang *et al.* (2012) assigned patients with mild-to-moderate Crohn’s disease (mean baseline 25(OH)D of 40.0 ± 25.0 nmol/L) to receive either 1000 IU/day for 2 weeks followed by a gradual increase in the dose until patients’ serum concentrations reached 100 nmol/L or 5000 IU/day for 24 weeks [79]. To achieve 25(OH)D > 100 nmol/L, the majority (78%) needed the larger dose of vitamin D supplement.

Antiepileptic drugs, glucocorticoids, orlistat and cholestyramine have been shown to affect circulating 25(OH)D [80]. Antiepileptic drugs and glucocorticoids have been shown to reduce 25(OH)D concentrations only when dietary sources of vitamin D (diet and supplements) and/or UV exposure is limited [80]. Orlistat, a lipase inhibitor, and cholestyramine, a bile acid sequestrant, may cause mal-absorption of fat and consequently impairment of vitamin D absorption. Treatment of obese adolescents with Orlistat for one month (*p* < 0.01) [81] and pre-pubertal children with familial hypercholesterolemia with cholestyramine for one year (*p* = 0.04) [82] were shown to significantly decrease 25(OH)D concentrations. However, other studies failed to show any difference in circulating 25(OH)D concentration between treatment and control group [83,84].

### 3.2. Treatment Strategy and Environmental Determinants

Apart from demographic and biological factors, are there extraneous factors which affect individual circulating 25(OH)D response to vitamin D supplementation (Table 2). These factors are determined either by strategies employed by researchers or health care providers such as type and dose of vitamin D supplements or by environment such as season.

#### 3.2.1. Type of Vitamin D; D_3_ *vs.* D_2_

It has been long believed that the two supplemental forms of vitamin D, D_3_ and D_2_, are equally effective in elevating or maintaining 25(OH)D concentrations. However, emerging evidence suggests that pharmacologic doses of vitamin D_2_ are not as potent as vitamin D_3_ (Table 2) [44,85,86,87,88,89], especially in the long-term [88] and when administered in bolus doses [90].

While Holick *et al.* (2008) [91] and Biancuzzo *et al.* (2013) [92] demonstrated that physiologic daily doses of vitamin D_2_ (1000 IU) are as effective as vitamin D_3_ (1000 IU), Armas *et al.* (2004) failed to show the equivalency of these isoforms [86]. The authors assigned healthy middle aged men to receive a single oral dose of 50,000 IU vitamin D_2_ or D_3_ [86]. After 28 days, the mean 25(OH)D concentration in the vitamin D_3_ group was higher by 22.0 nmol/L than vitamin D_2_ group. Based on the area under the curve (AUC), vitamin D_3_ was three-fold more potent than vitamin D_2_ (204.7 nmol/L *vs.* 150.5 nmol/L, respectively). Recruiting the same age group but both genders, Trang *et al.* (1998) also reported a larger increase in mean 25(OH)D in those receiving 4000 IU D_3_/day than those receiving 4000 IU D_2_/day for 14 days (23.3 ± 15.7 nmol/L *vs.* 13.7 ± 11.4 nmol/L (*P* = 0.03), respectively) [44]. Confirmed by a more recent trial, the average increase per 100 IU vitamin D_3_ and D_2_ was 1.45 nmol/L and 0.95 nmol/L, respectively [87]. Supplementation with vitamin D_2_ resulted in a significant decrease in mean 25(OH)D_3_ concentration, a finding confirmed by others [88,89,92].

Logan *et al.* (2013) showed that compared to vitamin D_3_, 25(OH)D_3_decreased [53 (95%CI, 45–61) nmol/L] and 25(OH)D_2_ increased in those receiving daily 1000 IU vitamin D_2_ for 25 weeks [88]. The absolute increase in 25(OH)D_2_ was 32 nmol/L per 1000 IU vitamin D_2_ daily. The decline in 25(OH)D_3_ could be explained by lower availability of substrate of vitamin D_3_ for hepatic hydroxylation. A drop in mean total 25(OH)D was reported in both vitamin D_3_ and placebo groups by approaching colder months which is consistent with what is known about the effect of season on 25(OH)D concentrations. Lehmann *et al.* (2013) [89] also reported a drop in mean 25(OH)D_3_ in both vitamin D_2_ and placebo groups, however, the decline in vitamin D_2_ group was 2.5 times more than that of the placebo group (approximately −20 *vs.* −8 nmol/L, respectively) suggesting other mechanisms.

The mechanistic pathways by which vitamin D_2_ may affect the metabolism of vitamin D_3_ are not clear. Some scientists suggest that physiologic doses of vitamin D_2_ does not interfere with vitamin D_3_ metabolism and may increase total 1,25(OH)_2_D (increase in 1,25(OH)_2_D_2_ accompanied by a slight decrease in 1,25(OH)_2_D_3_, mirroring the increase in 25(OH)D_2_ and decrease in 25(OH)D_3_, respectively) [91,92]. Armas *et al.*, on the other hand, suggested that the up regulation of mechanisms involved in vitamin D_2_ metabolism may lead to an increase in the degradation of 25(OH)D_3_ [86]. There is no evidence for such hypothesis. However, it is evident that 25-hydroxylase has higher affinity to vitamin D_3_ than vitamin D_2_ [93]. The rate by which vitamin D_3_ is hydroxylated in liver mitochondria is five times more than that of vitamin D_2_ (at a rate of 10 *vs.* 2 pmol/mg protein X minutes, respectively) [93]. While vitamin D_3_ is preferentially 25-hydroxylated vitamin D_2_ is 24-hydroxylated and deactivated [94]. This is not the case for vitamin D_3_; this molecule first undergoes 25-hydroxylation, then 24-hydroxylation and then an additional side chain oxidation to be biologically deactivated [95]. Houghton and Veith (2006) suggested that the higher affinity of hepatic hydroxylase, VDBP and VDR to vitamin D_3_ and its metabolites than vitamin D_2_ may explain the higher potency of vitamin D_3_ [95].

The lower affinity of 25(OH)D_2_ for VDBP than that of 25(OH)D_3_ results in a shorter half-life of 25(OH)D_2_ [96]. The shorter half-life along with a rapid catabolic rate of vitamin D_2_ metabolites might be more evident at the pharmacological doses. Furthermore, if 25(OH)D_2_ is not recognised the same as 25(OH)D_3_ by kidney and acts as an additional substrate, as was proposed by Biancuzzo *et al.* (2013), pharmacological doses of vitamin D_2_ may result in an increase in total 1,25(OH)_2_D. High concentrations of 1,25(OH)_2_D decrease the production and excretion of PTH [2] through a negative feedback loop, decrease the half-life of 25(OH)D [29,97], inhibit CYP27B1 activity and increase CYP24A1 (24-hydroxylase) activity and consequently the degradation of 25(OH)D [2].

It should be noted that despite the evidence (intervention and mechanistic studies) of differences in potency between the two isoforms, pharmacologic doses of vitamin D_2_, in clinical settings, have been proven to be effective in maintaining serum 25(OH)D above 50 nmol/L in people with vitamin D deficiency [98,99].

#### 3.2.2. Dosing Regimen

##### Dose

Evidence shows that higher doses are associated with smaller increases in 25(OH)D concentrations per unit of vitamin D compared with lower doses of vitamin D [13,46,100]. On the other hand, over the treatment period, higher doses administered either orally or intramuscularly result in greater increase in 25(OH)D concentrations and the increase is dose-dependent [13,15,40,46,47,52,58,87,100,101,102] (Table 2). Waterhouse *et al.* (2014) reported a dose-dependent increase in 25(OH)D concentration by using placebo or 30,000 and 60,000 IU vitamin D monthly and an incremental change of 2.2 nmol/L and 1.8 nmol/L per 100 IU vitamin D input in the 30,000 IU and 60,000 IU groups, respectively [46]. The larger doses of 100,000 IU/month [15] and 4000 IU/day [47] have been shown to be more potent in achieving 25(OH)D > 75 nmol/L than the lower doses of monthly 50,000 IU and daily 1000 and 2000 IU, respectively.

Hepatic hydroxylation is a saturable process and with an input above physiological norm of vitamin D, serum vitamin D concentration increases, and the reaction switches from the first order to zero order [103]. It is presumed that the excess vitamin D is stored in body fat as a native compound and is slowly released [60,103]. So, larger doses result in flatter slopes compared with the slopes for lower doses. This, therefore, may result in a longer apparent half-life of 25(OH)D. This mechanistic pathway may also explain the widely variable half-life reported to date in response to different doses of vitamin D supplementation.

**Table 2:** Treatment and environmental factors predicting circulating 25(OH)D response to vitamin D supplementation.

Study	Population Characteristics	Study Design/Duration/Groups	Relationship with			Description
			Type of Vitamin D	Dosing Regimen	Season	
Armas *et al.* (2004) [86]	Healthy men (*n* = 20)	Randomised control trial/28 days/Single oral dose of 50,000 IU D₂ or D₃	**Y**			The AUC to day 28 for D₃ and D₂ was 204.7 and 150.5 nmol/L, respectively.
Bacon *et al.* (2009) [43]	Elderly men and women (*n* = 63)	Randomised double blind trial/8 months/Single dose of 500,000 IU (loading dose), loading dose + monthly 50,000 IU or monthly 50,000 IU		**N**		The plateau was reached at one and 3–5 months in those receiving loading dose and monthly dose, respectively.
Barger-Lux *et al.* (1998) [40]	Healthy men (*n* = 116)	Open labelled trial/8 weeks/daily 1000, 10,000, or 50,000 IU D₃ or other vitamin D metabolites		**Y**		Stepwise increase in 25(OH)D concentrations (+29, +146, +643 nmol.L, respectively).
Biancuzzo *et al.* (2013) [92]	Healthy men and women (*n* = 34)	Randomised double blind placebo control trial/11 weeks/daily placebo or daily 1000 IU D₂ or daily 1000 IU D₃	Y*	**Y**		Greater increase was observed with vitamin D supplemented groups than placebo... * D_3_ group had a greater increase in 25(OH)D than D_2_ *(p* < 0.07).
Binkley *et al.* (2011) [87]	Healthy community-dwelling men and women (*n* = 64)	Randomised double blind placebo control trial/One year/daily 1600 IU D₂ or D₃ or monthly 50,000 IU D₂ or D₃ and matching placebos	**Y**	**Y**		The average increase per 100 IU D₂ and D₃ were 0.95 and 1.45 nmol/L, respectively
Blum *et al.* (2008) [49]	Healthy ambulatory men and women (*n* = 257)	Randomised placebo control trial/12 months/daily placebo or daily 700 IU D₃+ daily 500 mg calcium		**Y**	**Y**	Higher increase in 25(OH)D concentration in those receiving vitamin D supplement than placebo. Season was significantly associated with change in 25(OH)D levels (*p* < 0.01), but the direction was not reported.
Fu *et al.* (2009) [52]	Healthy adults (*n* = 98)	Open label un-blinded intervention trial/12 months/daily 600 or 4000 IU D₃		**Y**		Higher increase in 25(OH)D concentration in those receiving larger dose. Contribution of dose to overall variance was 22%.
Gallagher *et al.* (2012) [11]	Healthy postmenopausal women with vitamin D insufficiency (*n* = 163)	Randomised placebo control trial/12 months/daily placebo or daily 400, 800, 1600, 2400, 3200, 4000 or 4800 IU D₃+ daily 1200–1400 mg calcium		**Y**		A curvilinear dose-response relationship. Significant decrease in PTH levels with an increase in the dose of vitamin D₃.
Giusti *et al.* (2010) [54]	Healthy community-dwelling elderly women with secondary hyperparathyroidism and vitamin D deficiency (*n* = 59)	Randomised control trial/6 months/300,000 IU D₃/every 3 months or daily 1000 IU D₃ + daily 1500 mg calcium		**Y**		Mean increase was significantly lower in daily group compared to intermittent group (+34.3 ± 16.8 *vs.* +56.8 ± 29.5 nmol/L). Larger proportion of both treatment groups reached concentrations >75 nmol/L at 6 months compared to 3 months.
Harris *et al.* (2002) [39]	Healthy young and old men (*n* = 50)	Randomised control trail/8 weeks/daily 800 IU D₃		**Y**		Higher increase in 25(OH)D concentration in vitamin D supplemented groups than control group.
Hashemipour *et al.* (2010) [101]	Healthy men and women (*n* = 33)	Randomised double blind placebo control trial/4 months/Single dose of 0, 300,000 or 600,000 IU vitamin D₃ administered IM		**Y**		Circulating 25(OH)D increased significantly after 2 and 4 months but not after 2 weeks. Mean increase in 25(OH)D in 600,000 group was 2 times greater than in 300,000 group.
Heaney *et al.* (2003) [13]	Healthy men (*n* = 67)	Randomised placebo control trial/5 months//0, 1000, 5000, and 10,000 IU vitamin D₃/day		**Y**		Significant dose-dependent increase in 25(OH)D concentrations
Holick *et al.* (2008) [91]	Healthy multi-ethnic men and women (*n* = 68)	Randomised double blind trial/11 weeks/daily placebo or daily 1000 IU D_2_ or D_3_ or 500 IU D_2_ + 500 IU D_3_	**N**			Higher increase in 25(OH)D concentration in vitamin D groups than placebo group. D_2_ was potent as D_3_. Identical increase in 25(OH)D in all vitamin D groups.
Hollis and Wagner (2004) [102]	Healthy women (*n* = 18)	Randomised controlled trial/4 months/daily 1600 or 3400 IU D₂ + daily 400 IU D₃		**Y**		Higher increase in larger dose group.
Lehmann *et al.* (2013) [89]	Healthy adults (*n* = 107)	Randomised double blind placebo control trial/8 weeks/daily placebo or daily 2000 IU D_2_ or D_3_/	**Y**			Total 25(OH)D concentration was significantly different across groups (*p* < 0.001). Circulating 25(OH)D_3_ decreased in the placebo and vitamin D_2_ group (*p* < 0.001), but increased in the vitamin D_3_ group *(p* < 0.001).
Logan *et al.* (2013) [88]	Healthy men and women (*n* = 61)	Randomised double blind placebo control trial/25 weeks/0, 1000 IU/day D_2_ or 1000/day D₃	**Y**	**Y**		Total 25(OH)D concentration was significantly higher in vitamin D supplemented groups than placebo and in D_3_ group than D_2_.
Mazahery, Stonehouse, von Hurst (2015) [15]	Healthy premenopausal women (*n* = 61)	Randomised double blind placebo control trial/6 months/monthly placebo or monthly 50,000 or 100,000 IU D_3_		**Y**		Circulating 25(OH)D reached the plateau at 3 months. Larger proportion of women receiving 100,000 IU/month reached 25(OH)D concentrations >75 nmol/L at 6 months than those receiving 50,000 IU/months.
Nelson *et al.* (2009) [41]	Healthy premenopausal women (*n* = 112)	Randomised double blind placebo trial/21 weeks/daily placebo or daily 800 IU D_3_		**Y**	**Y**	Higher increase in vitamin D supplemented group than placebo. Starting the trial in winter was associated with a greater response. The magnitude of summer increase in 25(OH)D concentration was a significant predictor of the change.
Ng *et al.* (2014) [47]	Healthy adults (*n* = 292)	Randomised double blind placebo control trial/3 months/daily placebo or daily 1000, 2000 or 4000 IU D₃		**Y**		Larger proportion of subjects achieved 25(OH)D concentrations of 75 nmol/L or more; 3.7%, 37.0%, 63.8% and 90.4% in the placebo, 1000, 2000 and 4000 IU groups, respectively.
Nimitphong *et al.* (2013) [55]	Healthy adults (*n* = 39)	Un-blinded randomised control trial/3 months/daily 400 IU D_2_ or D_3_ + daily 675 mg calcium in al grups	**Y**			D_3_ tended to increase 25(OH)D concentration more than D_2_ (*p* = 0.08).
Putman *et al.* (2013) [56]	Healthy adolescents with vitamin D sufficiency (*n* = 53)	Double-blind, randomised clinical trial/11 weeks/daily 200 or 1000 IU D_3_		**N**	**Y**	Season of enrolment was a significant predictor.
Romagnoli *et al.* (2008) [85]	Women residence of nursing homes with vitamin D deficiency (*n* = 32)	Prospective randomised intervention/60 days/Single dose of 300,000 IU D_2_ or D_3_ administered orally or intramuscularly	**Y**	**Y**		Rapid and consistent increase with oral D_3_, but slow and gradual increase with both vitamins given intramuscularly. Based on the AUC, D_3_ was twice as potent as D₂.
Saadi *et al.* (2007) [38]	Healthy nulliparous and lactating women (*n* = 178)	Open-labelled, randomised, parallel group trial/3 months/daily 2000 IU D_2_ or monthly 60,000 IU D₂		**Y**		All women together, daily regimen was more effective than monthly regimen.
Talwar *et al.* (2007) [14]	Healthy postmenopausal women (*n* = 208)	Randomised placebo control trial/36 months /daily placebo or daily 800 IU D_3_ for the first 2 years and then daily 2000 IU D_3_ for the third year in the vitamin D group + daily 1200–1500 mg calcium in both groups		**Y**	**Y**	The slope was inversely associated with the dose used. Significant increase in 25(OH)D at 3 months and 27 months (+22%) in vitamin D group. The pre- and post-summer 25(OH)D concentrations were lower than the summer levels.
Trang *et al.* (1998) [44]	Healthy men and women (*n* = 72)	Randomised double blind trial/14 days/daily 4000 IU D_2_ or 4000 IU D_3_	**Y**			D_2_ and D_3_ increased mean 25(OH)D concentrations by 13.7 and 23.3 nmol/L, respectively.
Veith *et al.* (2001) [58]	Healthy men and women (*n* = 61)	Randomised intervention trial/2–5 months/daily 1000 or 4000 IU D_3_		**Y**		35% and 88% of participants in 1000 and 4000 groups achieved serum levels ≥75 nmol/L, respectively.
Waterhouse *et al.* (2014) [46]	Healthy older adults (*n* = 385)	Randomised double blind placebo control trial/one year/monthly placebo or monthly 30,000 or 60,000 IU D_3_		**Y**		Mean 25(OH)D increased to 78 ± 20 and 64 ± 17 nmol/L in the 60,000 and 30,000 IU groups, and the incremental change was 1.8 and 2.2 nmol/L per 100 IU vitamin D input, accordingly.
Zabihiyeganeh *et al.* (2013) [104]	Adults with 25(OH)D < 75 nmol/L (*n* = 79)	Open labelled randomised clinical trial/6 months/Single intramuscular 300,000 IU D_3_ or 6 divided oral doses (weekly 50,000 IU D_3_ for 4 weeks then monthly 50,000 IU).		**Y**		Change in 25(OH)D concentration was +90.0 ± 11.2 in the oral group and 58.8 ± 8.9 nmol/L in the intramuscular group at 3 months.
Zhao *et al.* (2012) [50]	Healthy postmenopausal women (*n* = 1063)	Randomised double blind placebo control trial/12 months/Placebo, 1100 IU/day vitamin D + 1400 mg/day calcium or 1400 mg calcium/day only.		**Y**	**Y**	Higher increase in 25(OH)D concentration in those receiving vitamin D than placebo. Starting the trial in winter was associated with a greater response

##### Frequency

The dosing frequency may influence the response to supplementation through its effect on compliance rate [45,87]. An intermittent regimen was more effective than a daily dose in ensuring a higher compliance rate; 80% and 100% in the daily and intermittent regimens, respectively [54]. Binkley *et al.* (2011) also reported a higher compliance rate in subjects receiving monthly regimen (50,000 IU; 99.4% in vitamin D_2_ and 98.9% in vitamin D_3_) than daily regimen (1600 IU; 95.4% in vitamin D₂ and 91.6% in vitamin D_3_) [87].

##### Route

Vitamin D supplements administered intramuscularly (IM) are often given in bolus dosages, and are useful for patients with absorption disorders and with low compliance and in areas where oral supplements are not available. However, there are some concerns about the safety [105] and effectiveness of vitamin D administered IM [85,104]. Zabihiyeganeh *et al.* (2013) showed that 25(OH)D response to supplementation was better in oral than IM form (+90.0 ± 11.2 nmol/L *vs.* +58.8 ± 8.9 nmol/L, respectively; *p* = 0.03) [104]. It should be noted that despite having the same accumulative dose of 300,000 IU vitamin D, the dosing regimens were completely different across treatment groups; the IM group received a single 300,000 IU vitamin D but the oral group received weekly 50,000 IU vitamin D for four weeks and then monthly for two months. When subjects were followed up after 6 months, there was no significant difference in 25(OH)D between the oral and IM groups, a finding confirmed by others [85]. However, the proportion of subjects attaining 25(OH)D levels ≥75 nmol/L (65.0% *vs.* 43.6%, respectively, *p* = 0.06) [85] and the mean 25(OH)D concentrations [104] at the follow-up were marginally higher in the oral group than IM group. The authors suggested that a longer period would be needed to observe significant changes in serum 25(OH)D concentrations when vitamin D supplements are administered IM.

##### Duration

Several trials have shown that 25(OH)D response to vitamin D supplementation peaks at three-months [14,15,104], while others suggest that 6 months is needed [54] (Table 2). Talwar *et al.* (2007) assigned healthy post-menopausal women to receive either daily 800 IU for two years followed by daily 2000 IU vitamin D for one year or placebo through the entire study period [14]. Mean 25(OH)D concentration in the active arm increased significantly at three months and 27 months (three months after the initiation of the second dose), but decreased at 24 and 36 months indicating that 25(OH)D concentrations reached the peak after three months of supplementation.

In another study, however, 25(OH)D concentrations increased and PTH levels decreased significantly at six months compared with three months (*p* < 0.001) [54]. Furthermore, the proportion of cases reaching 25(OH)D levels >75 nmol/L was higher at 6 months than 3 months [15,54]. Unrelated to the change in 25(OH)D concentrations, bone turn-over markers decreased significantly at two different time points but the improvement was greater at six months. Accordingly, six months of vitamin D supplementation, but not more, would be enough to see the maximum biological response as in 25(OH)D response. Based on the dose response curve, the slope of six months did not differ significantly from that of 12 months [53].

#### 3.2.3. Season

Seasonal effect on 25(OH)D concentrations has been reported in several trials. Circulating 25(OH)D concentration has been frequently reported to be lower in winter than summer months due to the vitamin D input from sun exposure [106]. The seasonal impact on response to vitamin D supplementation has been recently reported by several studies [14,41,49,50,56] (Table 2). Compared to the hotter months, when vitamin D supplementation is initiated in colder months, the incremental change in circulating 25(OH)D concentration is higher. The effect of season is partly explained by the effect of baseline status on change in response to supplementation discussed in Section 3.1.1. Lower 25(OH)D concentrations are associated with higher PTH levels in circulation [106], and higher PTH levels, on the other hand, may increase hepatic 25(OH)D clearance [107]. Even though the baseline 25(OH)D concentration was controlled in linear regression analysis, the effect of season remained significant in a study by Zhao *et al.* (2012) [50]. Holick (2007), in his review, suggested an endogenous mechanism by which people are protected from vitamin D intoxication during the long-term sun exposure in summer months [2]. Excessive sun exposure degrades both pre-vitamin D_3_ and vitamin D_3_ and converts them into inactive photoproducts including lumisterol, tachysterol and 7-dihydroxy cholesterol [108].

## 4. Conclusions

The relationship between 25(OH)D concentration and vitamin D supplementation is not straightforward and is influenced by a large number of factors. Some of these factors such as basal 25(OH)D concentration are well documented. Evidence is emerging for others such as BMI/body fat% and season, while for calcium intake, dietary fat content and composition, and genetics the evidence is either mixed or in its infancy. The mechanisms by which these factors may affect the response are not well understood. Accordingly, there is an urgent need for more well-designed studies: (1) to establish the significance of these factors; (2) to identify other unknown factors; (3) to determine the mechanistic pathways by which these factors may exert their roles and (4) to strengthen our knowledge and understanding to inform the dose of supplementation required. It should be noted that increasing 25(OH)D concentration alone is not meaningful if it is not accompanied by improved clinical outcomes, or at least biomarkers. Clinical trials investigating the impact of improving vitamin D status on various health and disease outcomes are also warranted. Finally, chronic diseases are multifactorial in origin and many variables that contribute to the development of these diseases, such as unhealthy dietary habits, aging and physical inactivity, are risk factors for vitamin D deficiency [109]. Therefore even if widespread vitamin D status is optimized, various chronic diseases will continue to occur, but the risk will be lower.
